# Effects of kefir consumption on gut microbiota and health outcomes in women with polycystic ovary syndrome

**DOI:** 10.1002/fsn3.4212

**Published:** 2024-05-15

**Authors:** Merve Esra Çıtar Dazıroğlu, Nilüfer Acar Tek, Münire Funda Cevher Akdulum, Canan Yılmaz, Ayşe Meltem Yalınay

**Affiliations:** ^1^ Department of Nutrition and Dietetics Gazi University Ankara Turkey; ^2^ Department of Gynecology and Obstetrics Gazi University Ankara Turkey; ^3^ Department of Medical Biochemistry Gazi University Ankara Turkey; ^4^ Department of Medical Microbiology Gazi University Ankara Turkey

**Keywords:** health, kefir, microbiota, polycystic ovary syndrome

## Abstract

Polycystic Ovary Syndrome (PCOS), which is common among women of reproductive age, is characterized by low‐grade chronic inflammation and is associated with several health problems and dysbiosis. Kefir has been shown to have many beneficial health effects; however, its effect on PCOS is unknown. This study aimed to examine the effect of kefir on the intestinal microbiota and health outcomes in PCOS. In this intervention study, 17 women with PCOS consumed 250 mL/day of kefir (containing *Lactobacillus kefiranofaciens* subsp. *kefiranofaciens*, *Lactobacillus kefiranofaciens* subsp. *kefirgranum*, *Lactobacillus kefiri*, *Lactobacillus acidophilus*, *Lactobacillus parakefiri*, *Lactobacillus bulgaricus*, *Lactobacillus reuteri*, *Lactobacillus casei*, *Lactobacillus fermentum*, *Lactobacillus helveticus*, *Lactococcus lactis*, *Leuconostoc mesentereoides*, *Bifidobacterium bifidum*, *Streptococcus thermophilus*, *Kluyveromyces marxianus*, *Kluyveromyces lactis*, *Acetobacter pasteurianus*, and *Saccharomyces cerevisiae*) for 8 weeks. Food consumption and physical activity records, anthropometrical measurements, quality of life, and fecal and blood samples were taken at the study's beginning and end. Quality of life in mental health (58.8 ± 15.08; 64.0 ± 15.23, respectively) and physical function (95.00 and 100.00, respectively) categories showed a significant increase after kefir intervention (*p* < .05). Additionally, Interleukin‐6 (IL‐6), one of the inflammatory cytokines, significantly decreased (174.00 and 109.10 ng/L, respectively) (*p* < .05). The intestinal barrier permeability was evaluated with zonulin, and no significant change was observed. Gut microbiota analysis showed that while the relative abundance of the class Bacilli and genus *Lactococcus* significantly increased, the genus *Holdemania* decreased with kefir consumption (*p* < .05). In conclusion, kefir appears to be beneficial for improving the microbiota and some health outcomes, like reducing inflammation and improving quality of life in PCOS. Therefore, kefir may be useful in the treatment of PCOS.

## Graphical abstract

Polycystic ovary syndrome (PCOS), characterized by chronic low‐grade inflammation, has many complications. Consuming 250 mL/day of kefir for 8 weeks increased the quality of life and reduced IL‐6 levels. In addition, while no significant change was observed in intestinal barrier permeability with kefir consumption, some positive changes were determined in the intestinal microbiota.

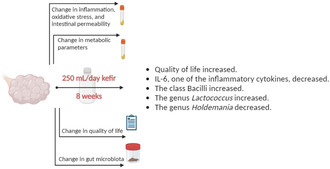

## 1 INTRODUCTION

Polycystic Ovarian Syndrome (PCOS) is a complex endocrine disorder affecting women of reproductive age, characterized by such clinical manifestations as menstrual irregularities, hyperandrogenism, and polycystic ovaries (Hajam et al., 2024). There is no globally accepted treatment for PCOS. It is essential that treatment be addressed at an individual level, depending on women's symptoms, and shaped within the framework of their needs (Escobar‐Morreale, 2018). Because PCOS affects women for life, when managed well, there is only remission rather than full recovery. In PCOS, although different treatment priorities and targets are determined according to the course of the disease, lifestyle interventions take priority. Related to this, it is a priority for PCOS to be careful with their nutrition in treatment (Che et al., 2023).

Along with the existence of high inflammatory cytokines, C‐reactive protein (CRP) levels, and increased leukocyte count, PCOS is associated with low‐grade chronic inflammation (Diamanti‐Kandarakis et al., 2006). In addition to inflammation, oxidative stress also appears to be increased in PCOS (Gao et al., 2023), with lower serum levels of Total Antioxidant Status (TAS), antioxidants like vitamin E and vitamin C, and higher levels of oxidative stress markers like malondialdehyde (MDA), paraoxonase‐1 (PON1), xanthine oxidase, uric acid, advanced oxidation protein products (AOPP), and advanced glycation end products (AGEs) (Azim et al., 2024; Bahreiny et al., 2024; Fenkci et al., 2003; Mohammadi, 2019). Increased homocysteine levels in PCOS (Sharma et al., 2024) also contribute to oxidative stress (Faverzani et al., 2017). Increased oxidative stress and decreased antioxidant capacity (Fenkci et al., 2003) contribute to the increased risk of many reproductive and metabolic health problems in PCOS. These include dyslipidemia, type 2 diabetes, hypertension, insulin resistance, obesity, cardiovascular diseases, metabolic syndrome, and infertility (Di Lorenzo et al., 2023; Fenkci et al., 2003; Thackray, 2019). Related to all these, there are alterations in the intestinal microbiota in PCOS, and it is stated that the diversity of the intestinal microbiota changes (Sun et al., 2023).

Hyperandrogenism, hyperinsulinemia, and inflammation are the main factors affecting the microbiota in PCOS (He & Li, 2020; Singh et al., 2023). It is known that there are differences in the microbiota composition of PCOS compared to healthy controls, a decrease in biodiversity, and changes in specific bacterial taxa in the intestinal microbiome of PCOS (Guo et al., 2022). It has been shown in multiple studies to date that alpha diversity decreases in PCOS (Insenser et al., 2018; Jobira et al., 2020; Liu et al., 2017). It is possible to see different results in studies on the microbiota composition of women with PCOS. For example, regarding the Firmicutes and Bacteroidetes phyla, a decreased or lower Firmicutes/Bacteroides ratio (Zhou, Ni, Yu, et al., 2020) or the lower phylum Bacteroidetes and the similar phylum Firmicutes have also been reported (Jobira et al., 2020). Although dysbiosis in the intestinal microbiota has been shown to be important in PCOS (Insenser et al., 2018; Jobira et al., 2020; Liu et al., 2017), it is stated that further studies are needed due to inconsistent results in some studies showing that the abundance of some bacteria increased, and in others, these bacteria decreased (Yurtdaş & Akdevelioğlu, 2020).

Kefir, on the other hand, is a fermented dairy product that has shown similar effects in various disease groups to date and can regulate the homeostasis of the organism by directly affecting the gut‐brain axis (Peluzio et al., 2021). The positive effect of kefir is related to the various bioactive compounds produced during fermentation, including bioactive peptides, exopolysaccharides, organic acids, and bacteriocins, and the diverse microbiota it contains (González‐Orozco et al., 2022). Kefir can be produced industrially or traditionally. Traditional kefir is produced using grain, and kefir grains are a complex consortium of yeast and bacteria with a symbiotic relationship. Traditional kefir has higher antioxidant content (McGovern et al., 2024) and higher microbial diversity (Biçer et al., 2021). An in vivo study has shown that the microbiota of kefir produced by natural kefir grain causes boosted immunomodulator properties as compared to kefir produced using starter culture, as is typical in commercial kefir (Davras et al., 2018). On the other hand, it is stated that by‐products from dairy processing are a source of waste, and this negatively affects the environment. Therefore, finding applications for by‐products is significant for reducing waste and contributing to sustainability (McGovern et al., 2024). Encapsulation technology from probiotics in sustainable food production is important (Agriopoulou et al., 2023). Encapsulated probiotics may also show better viability and stability in kefir during storage (Afzaal et al., 2022; Agriopoulou et al., 2023).

Based on all this, although the positive effects of kefir on health in different diseases have been shown so far, there is no study examining the effects of kefir consumption on PCOS. Therefore, this study was conducted to evaluate the effects of kefir consumption on intestinal microbiota modulation, and some health outcomes such as quality of life, oxidative stress, and inflammation biomarkers.

## 2 MATERIALS AND METHODS

### 2.1 Subjects

This was an intervention study that was executed from October 2022 to March 2023 among the 17 women with PCOS (according to Rotterdam criteria, which includes suffering from at least two of the following symptoms: oligo‐ or anovulation, clinical and/or biochemical signs of hyperandrogenism, polycystic ovaries, and exclusion of other etiologies) in Gazi University Hospital, Department of Gynecology and Obstetrics. Women diagnosed with PCOS aged between 18 and 40 years and whose Body Mass Index (BMI) values were between 18.5 and 29.99 kg/m^2^ were included in the study. The exclusion criteria were as follows: pregnancy or breastfeeding, smoking, alcohol consumption, having endocrine diseases, taking lipid‐lowering drugs or blood pressure regulating medication, taking antibiotics or medications related to PCOS treatment or vitamin–mineral or probiotics within 1 month prior to study inclusion.

### 2.2 Study protocol

In this study, individuals were followed for 8 weeks, and during this period, they were asked to consume 250 mL/day of kefir and maintain their routine nutrition and physical activity habits. The kefir consumed in the course of the study was delivered to the participants by the researcher in seven bottles every week, thus keeping in touch with the participants every week and at the same time aiming to preserve the microbial composition of the kefir as much as possible. Before giving kefir to the participants, it was kept in the refrigerator after receiving it from the company, and care was taken not to disrupt the cold chain.

**FIGURE 1:**
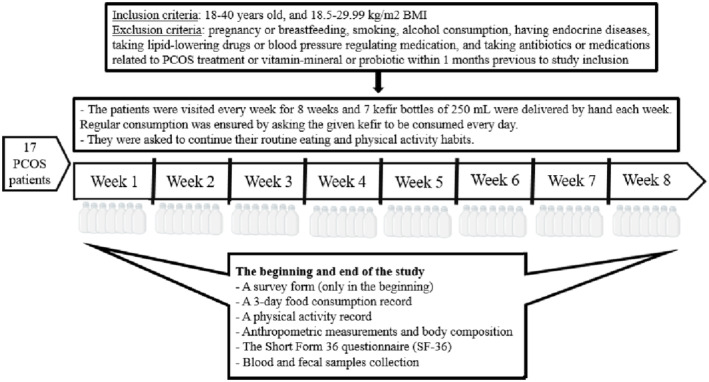
Timeline of the study.

The visit at the beginning of the study included applying a survey form, assessing a 3‐day food consumption record, a physical activity record, the SF‐36 quality‐of‐life scale, anthropometric measurements, body composition, and collecting the initial blood and fecal samples. These were repeated at the end of the study (except for the survey). The timeline of the study is summarized in Figure 1.

### 2.3 Kefir used in the study

In this study, traditional kefir produced with grains by Danem, Inc. (kefirdanem.com, Suleyman Demirel University Technopark, Isparta, Turkey) was used. This kefir was preferred for reasons such as its high diversity of microorganisms and the fact that its content information is shared by the company. The composition of the microbial population can be given as follows: *Lactobacillus kefiranofaciens* subsp. *kefiranofaciens*, *Lactobacillus kefiranofaciens* subsp. *kefirgranum*, *Lactobacillus kefiri*, *Lactobacillus acidophilus*, *Lactobacillus parakefiri*, *Lactobacillus bulgaricus*, *Lactobacillus reuteri*, *Lactobacillus casei*, *Lactobacillus fermentum*, *Lactobacillus helveticus*, *Lactococcus lactis*, *Leuconostoc mesentereoides*, *Bifidobacterium bifidum*, *Streptococcus thermophilus*, *Kluyveromyces marxianus*, *Kluyveromyces lactis*, *Acetobacter pasteurianus*, and *Saccharomyces cerevisiae*. The count of the microbial population, on the other hand, can be given as follows: *Lactobacillus* spp.: 10.54 log kob/mL; *Lactococcus* spp.: 10.62 log kob/mL; and yeast: 2.69 log kob/mL (Güzel‐Seydim & Kök Taş, 2024). Analyses regarding the microorganism content of the kefir used in the research are carried out regularly by the company mentioned above, and the results of the analyses are similar to each other. Various study results regarding kefir have also been published by the company's founders (Gökırmaklı & Güzel‐Seydim, 2022; Kök Taş et al., 2012).

### 2.4 Anthropometrical measurements and body composition

Individuals' body weight measurements were measured using a Tanita BC 532 brand device, and height measurements were measured using a Leicester brand stadiometer. Body compositions were taken using the Inbody S10 bioelectrical impedance analysis device. As a result of the measurement of the device, individuals' total body water (L), intracellular and extracellular fluid (L), body fat percentage (%), body cell mass (kg), fat mass (kg), skeletal muscle mass (kg), and lean body mass (kg) values were recorded.

### 2.5 The short‐form 36 questionnaire (SF‐36)

For the purpose of evaluating the quality of life of the participants, SF‐36 was administered twice in total, at the beginning and end of the 8‐week follow‐up period. The scale developed by Ware and Sherbourne (1992) has 8 subscales (social function, physical function, pain, physical role limitation, emotional role limitation, mental health, vitality (energy), and general perception of health) and 36 items. The scale gives a total score for every subscale separately, and the scores vary between 0 and 100. Higher scores on the scale indicate better health status, while lower scores reflect poorer health status (Koçyigit et al., 1999; Ware & Sherbourne, 1992).

### 2.6 Biochemical assessment

Blood samples were taken after an 8‐h overnight fast at the baseline (week 0) and the end of the study (week 8). Serum fasting glucose, insulin, triglycerides, low‐density lipoprotein (LDL‐C), high‐density lipoprotein (HDL‐C), total cholesterol, and C‐reactive protein (CRP) from blood samples were analyzed according to conventional laboratory standard methods. Another tube of blood sample was centrifuged, and then stored at −80°C until further analysis. Using the BT LAB kit from this blood, IL‐6, TNF‐α, and zonulin were studied with an enzyme‐linked immunosorbent assay (ELISA). Total Oxidant Status (TOS) and Total Antioxidant Status (TAS) analyses were performed using the Rel Assay kit. MDA levels, on the other hand, were measured using the thiobarbituric acid test method. Homeostatic Model Assessment (HOMA‐IR) was computed with the formula “the fasting insulin level (mU/L) × fasting plasma glucose (mg/dL)/405.” The oxidative stress index (OSI), an indicator of the degree of oxidative stress, is computed by dividing the TOS value by TAS (Harma & Erel, 2003; Kosecik et al., 2005).

### 2.7 Fecal DNA extraction, sequencing, and bioinformatic analysis

The participants' stool samples were collected from different parts of the stool in sterile tubes twice, at the beginning and the end of the study, and were stored at −80°C until analyzed. In all images regarding microbiota analysis in the study, Before Treatment (BT) refers to the period before the intervention, and Post‐Treatment (PT) refers to the period after the intervention.

The DiaRex® Stool Genomic DNA Extraction Kit was used for stool samples. After adding 250 μL Stool Lysis (SLD) solution onto an average of 25–50 mg stool sample, 10 zircon beads and 15 mg glass were added, and the application was made in the homogenizer device at 4000 rpm for 2 × 20 s. After homogenization, 25 μL of Proteinase K (PKD) was added. Next, it was incubated at 56°C for 60 min. After incubation, the entire content was centrifuged at 5000 *g* for 5 min, and the supernatant was transferred to a new tube. Afterwards, 200 μL of Lysis (LBD) solution was added to the supernatant and incubated at 70°C for 10 min. After incubation, 250 μL of absolute ethanol was added to the lysate, and the entire content was transferred to the column. The column was centrifuged at 8000 *g* × 1 min and then transferred to a new tube. Following this, after washing in accordance with the kit protocol, 100 μL of elution (EBD) was added and incubated for 2 min. Genomic DNA was obtained by centrifuging at 8000 *g* × 1 min.

The extracted DNA was amplified with 16S V3‐V4 314F‐860R primer sets. Library preparation, on the other hand, was handled with the Nextera XT DNA library preparation kit and indexes (Illumina). Pooled libraries cleaned by specific size selection were performed according to the manufacturer's protocol (AMPure XP, Beckman Coulter). Following library preparation, the MiSeq (Illumina) instrument was used to run sequencing.

Pair‐end Illumina reads (2 × 250) were significant to the QIIME2 environment (Bolyen et al., 2019). All of the samples have more than 100× sequence depth, and no samples were removed from the study. Chimera detection and quality clipping were implemented through the QIIME2 Dada2 pipeline (via q2‐dada2) (Callahan et al., 2016). Amplicon Sequence Variants (ASV) were created by excluding parts with quality scores below 30. Taxonomic tables were created by mapping the obtained ASVs with the Silva 138 (https://www.arb‐silva.de/documentation/release‐138/) database (Schloss, 2021; Werner et al., 2012). For biostatistical analyses and data visualization, files created with QIIME2 were processed using the R 4.1 programming language in R Studio (McMurdie & Holmes, 2013; R Core Team, 2013). Alpha diversity assessment was interpreted using Shannon, Simpson, and Chao1. The *p*‐values between groups were calculated with the Kruskal–Wallis test (Kruskal & Wallis, 1952). Beta diversity analysis was calculated based on unweighted and weighted unifracs (Lozupone et al., 2007; Lozupone & Knight, 2005). Linear Discriminant Analysis (LDA) Effect Size (LEfSe) was made between groups to indicate statistically significant taxonomies (Segata et al., 2011). In the LEfSe analysis, LDA scores above 2 were considered significant.

### 2.8 Statistical analysis

The data obtained from the study were evaluated and interpreted using the SPSS (Statistical Package for the Social Sciences) 22.0 package program. While qualitative variables are given as numbers (S) and percentages (%), quantitative variables are expressed as mean ($\bar{X}$), standard deviation (SD), and median (min‐max). The Shapiro–Wilk test was used to evaluate compliance with normal distribution, and when comparing two dependent groups, the paired samples *t*‐test was used for those with a normal distribution and the Wilcoxon test for those not suitable for a normal distribution. In all statistical evaluations, *p* < .05 was considered statistically significant.

### 2.9 Ethical approval

The study protocol was approved by the Ethical Committee of the Gazi University of Ankara/Turkey (February 21, 2022, Decision no: 156). In addition, participants signed a consent form stating that they participated voluntarily.

## 3 RESULTS

### 3.1 Characteristics of study participants

The average age of the participants is 24.7 ± 5.44 years, and 64.7% are single (not shown in the table). There were no significant differences in body composition, dietary intake, anthropometric measurements, and physical activity levels before and post‐treatment (Table 1).

**TABLE 1:** Some characteristics of the participants at the beginning and end of the study.

Variables	Before treatment	Post‐treatment	*t*	*p*‐Value
	$\bar{X}$ ± ss	$\bar{X}$ ± ss		
Energy and macronutrients				
Energy (kcal)	1568.6 ± 242.92	1585.3 ± 249.71	−0.478	.639
Carbohydrate (%)	42.4 ± 4.86	43.6 ± 6.24	−0.615	.547
Protein (%)	14.5 ± 1.94	15.4 ± 2.69	−0.919	.372
Fat (%)	43.2 ± 4.06	41.2 ± 6.46	1.163	.262
Anthropometric measurements and body composition				
Body weight (kg)	59.9 ± 8.78	60.2 ± 8.79	−0.923	.370
Body mass index (kg/m^2^)	23.0 ± 2.73	23.1 ± 2.62	−0.846	.410
Waist circumference (cm)	76.8 ± 7.17	76.9 ± 6.76	−0.198	.846
Hip circumference (cm)	98.8 ± 5.89	98.2 ± 5.97	1.814	.089
Neck circumference (cm)	31.4 ± 1.63	31.1 ± 1.55	1.186	.253
Waist/hip ratio	0.8 ± 0.05	0.8 ± 0.04	−1.482	.158
Waist/height ratio	0.5 ± 0.04	0.5 ± 0.04	−0.206	.839
Lean body mass (kg)	42.2 ± 5.47	42.8 ± 5.45	−0.796	.438
Body fat mass (kg)	17.7 ± 6.23	17.4 ± 5.29	0.442	.665
Body fat percentage (%)	29.0 ± 7.62	28.5 ± 5.88	0.408	.689
Skeletal muscle mass (kg)	23.2 ± 3.25	23.6 ± 3.33	−0.838	.415
Body cell mass (kg)	27.7 ± 3.59	28.1 ± 3.65	−0.919	.372
Total body water (L)	30.9 ± 4.01	31.2 ± 3.95	−0.726	.478
Intracellular fluid (L)	19.3 ± 2.51	19.6 ± 2.54	−0.872	.396
Extracellular fluid (L)	11.5 ± 1.51	11.6 ± 1.42	−0.446	.661
Physical activity level	1.6 ± 0.13	1.6 ± 0.12	−0.969	.347

*Note*: Paired samples *t* test.

### 3.2 Change in quality of life

Regarding quality of life, a statistically significant increase was determined in the participants' mental health and physical function scores (*p* < .05) (Table 2).

**TABLE 2:** Participants' quality‐of‐life scores at the beginning and end of the study.

Variables	Before treatment		Post‐treatment		*t* ^a^	*Z* ^b^	*p*‐Value
	$\bar{X}$ ± ss	Median (min‐max)	$\bar{X}$ ± ss	Median (min‐max)			
Physical function	92.1 ± 10.01	95.00 (75.00–100.00)	96.2 ± 6.97	100.00 (75.00–100.00)		−2.226	.026*
Social function	70.6 ± 24.98	62.50 (25.00–100.00)	83.1 ± 20.22	87.50 (37.50–100.00)		−1.852	.064
Pain	60.6 ± 32.26	72.00 (0–100.00)	69.5 ± 31.90	84.00 (0–100.00)		−1.781	.075
Vitality (energy)	49.4 ± 16.38	50.00 (20.00–75.00)	54.4 ± 11.58	55.00 (25.00–70.00)	−1.404		.179
Emotional role limitation	39.2 ± 47.49	0 (0–100.00)	60.8 ± 44.46	66.67 (0–100.00)		−1.852	.064
Physical role limitations	70.6 ± 42.61	100.00 (0–100.00)	88.2 ± 25.18	100.00 (0–100.00)		−1.561	.119
Mental health	58.8 ± 15.08	60.00 (36.00–80.00)	64.0 ± 15.23	68.00 (32.00–84.00)	−2.139		.048*
General perceptions of health	54.6 ± 21.16	52.00 (20.00–87.00)	61.4 ± 22.00	67.00 (15.00–92.00)	−1.823		.087

^a^ Paired samples *t* test.

^b^ Wilcoxon test.

* *p* < .05.

### 3.3 Changes in metabolic status, biomarkers of oxidative stress and inflammation, and zonulin levels

Metabolic parameters, markers of oxidative stress and inflammation, and zonulin levels of the participants are summarized in Table 3. While there was no significant difference for metabolic parameters, the IL‐6 level, one of the inflammation markers, decreased significantly (*p* < .05).

**TABLE 3:** Biochemical parameters of participants at the beginning and end of the study.

Variables	Before treatment		Post‐treatment		*t* ^a^	*Z* ^b^	*p*‐Value
	$\bar{X}$ ± ss	Median (min‐max)	$\bar{X}$ ± ss	Median (min‐max)			
Metabolic parameters							
Serum fasting glucose (mg/dL)	83.5 ± 4.67	83.00 (75.00–93.00)	81.4 ± 7.42	82.00 (67.00–98.00)	1.251		.229
Serum fasting insulin (mU/L)	7.2 ± 2.39	7.90 (3.10–11.25)	7.1 ± 2.89	7.04 (2.30–14.24)	0.142		.889
HOMA‐IR	1.5 ± 0.52	1.60 (0.57–2.32)	1.4 ± 0.55	1.40 (0.46–2.39)	0.449		.624
Triglyceride (mg/dL)	67.3 ± 19.23	66.00 (44.00–100.00)	79.0 ± 24.09	71.00 (52.00–129.00)	−1.958		.068
HDL‐cholesterol (mg/dL)	59.9 ± 11.42	55.00 (43.00–78.00)	58.9 ± 13.25	58.40 (41.70–80.20)	0.527		.605
LDL‐cholesterol (mg/dL)	112.3 ± 26.05	114.00 (70.00–156.00)	103.9 ± 26.12	109.00 (49.00–143.00)	1.915		.073
VLDL‐cholesterol (mg/dL)	13.5 ± 3.91	13.00 (9.00–20.00)	15.7 ± 4.97	14.00 (10.00–26.00)		−1.661	.097
Total cholesterol (mg/dL)	185.6 ± 28.82	188.00 (134.00–243.00)	178.5 ± 34.29	171.00 (105.00–243.00)	1.336		.200
Markers of oxidative stress and inflammation							
IL‐6 (ng/L)	291.3 ± 262.09	174.00 (54.46–1011.00)	194.7 ± 247.28	109.10 (26.14–914.70)		−3.053	.002*
TNF‐α (ng/L)	575.1 ± 734.75	318.20 (81.22–3012.00)	504.5 ± 621.66	293.40 (53.12–2309.00)		−1.207	.227
CRP (mg/L)	2.6 ± 1.64	2.01 (1.06–7.39)	2.9 ± 2.13	2.26 (1.17–9.94)		−0.971	.332
MDA (nmol/L)	5.8 ± 1.91	5.56 (3.08–9.66)	6.3 ± 3.99	4.69 (2.71–16.01)		−0.544	.586
TAS (mmol/L)	1.5 ± 0.15	1.51 (1.39–1.92)	1.5 ± 0.18	1.56 (1.21–1.84)		−0.284	.776
TOS (μmol/L)	4.0 ± 1.35	3.93 (2.03–7.70)	3.8 ± 2.03	3.08 (1.91–8.30)		−1.349	.117
OSI	0.3 ± 0.09	0.25 (0.15–0.49)	0.3 ± 0.17	0.20 (0.13–0.69)		−1.396	.163
Intestinal permeability							
Zonulin (ng/mL)	46.0 ± 59.13	21.00 (4.47–250.00)	43.6 ± 60.19	20.69 (3.95–236.20)		−1.065	.287

^a^ Paired samples *t* test.

^b^ Wilcoxon test.

* *p* < .05.

### 3.4 Changes in gut microbiota composition

**FIGURE 2:**
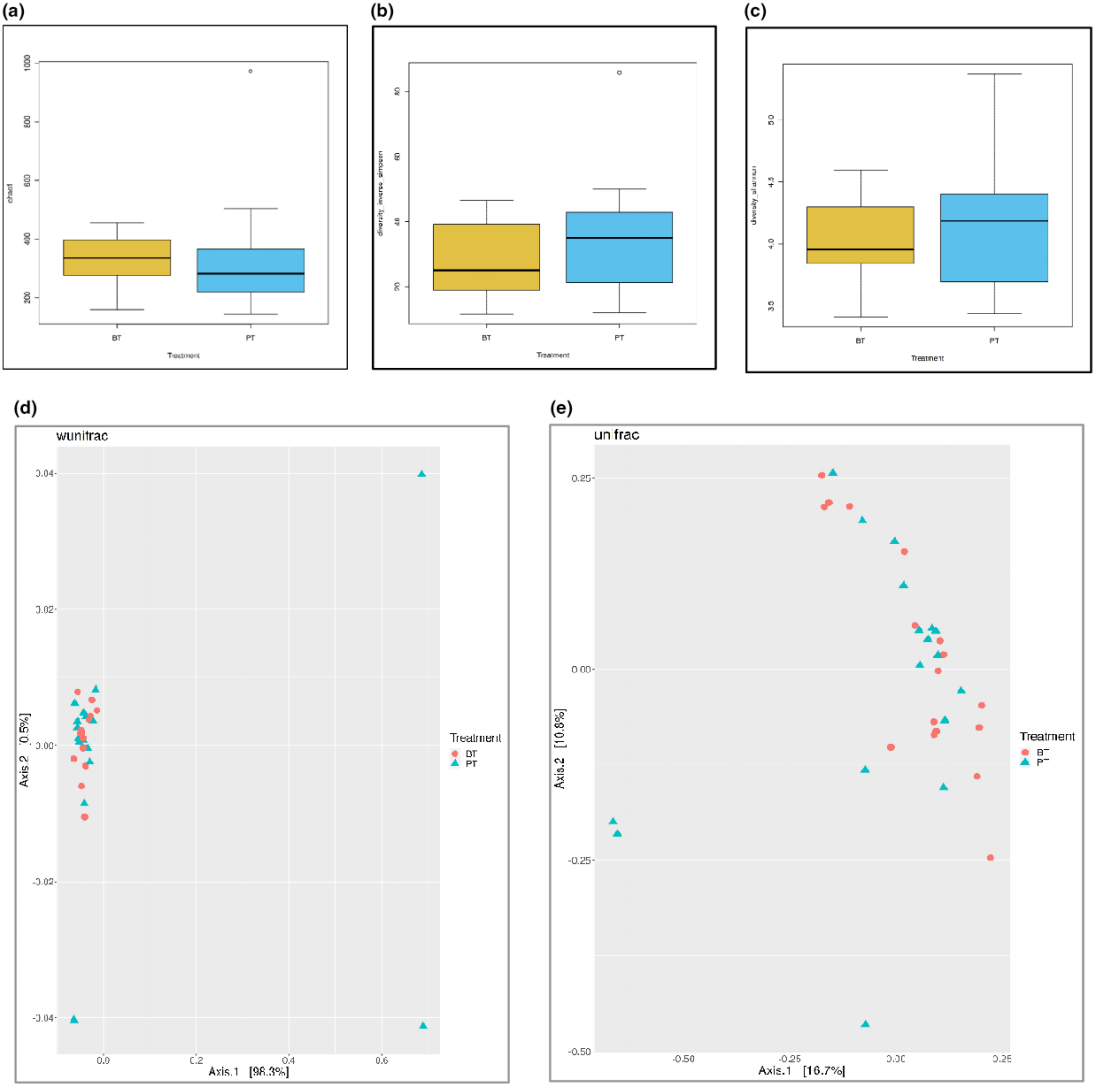
Box plots of the alpha diversity and the PCoA plots of the weighted and unweighted UniFrac distances for the before and post treatment (a: Chaol, b: Simpson index, c: Shannon index, d: Weighted UniFrac, e: Unweighted UniFrac) (BT, before treatment; PT, post‐treatment).

Changes in beta and alpha diversity indices are presented in Figure 2. Accordingly, no significant change was observed in the beta and alpha diversity indexes.

**FIGURE 3:**
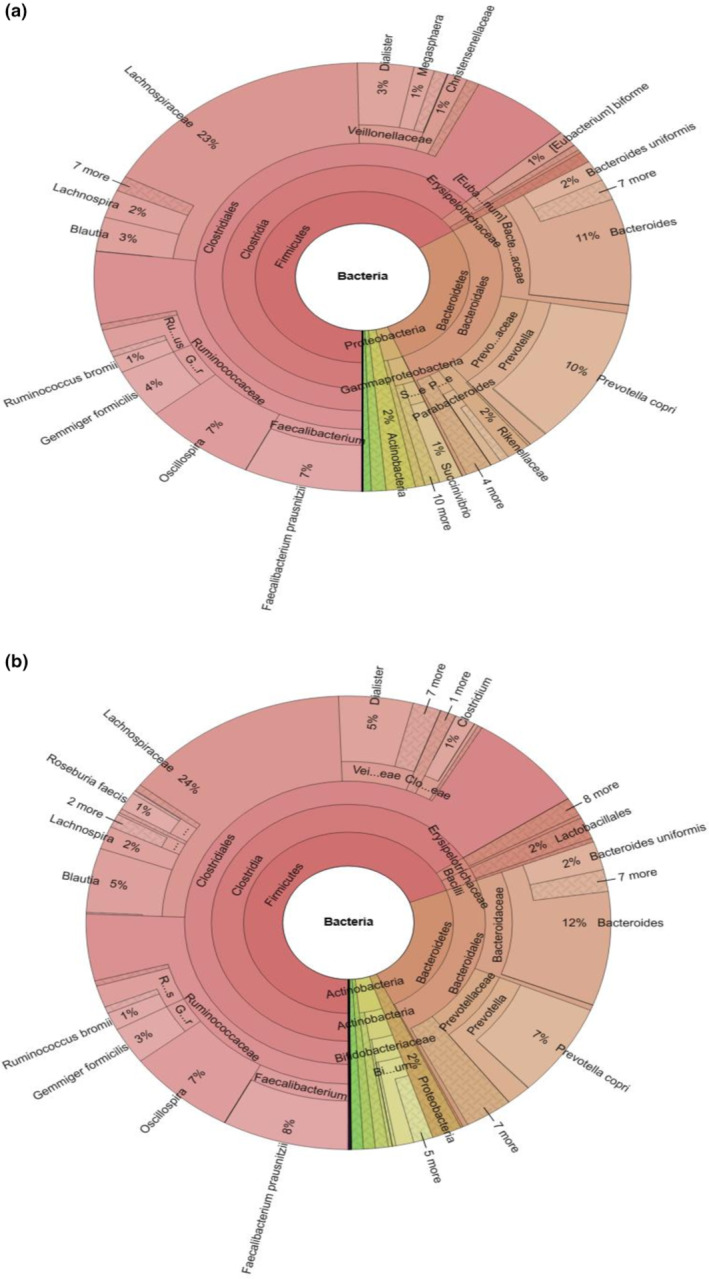
Krona displays. (a: Before treatment Krona display, b: Post‐treatment Krona display). Krona displays abundance and hierarchy simultaneously using a radial space‐filling display. The Krona chart features a red‐green color gradient signifying average e‐values of BLAST hits within each taxon (Ondov et al., 2011).

Krona displays of the participants before and after the intervention are shown in Figure 3. Accordingly, it was determined that the most dominant species before the intervention belonged to the phyla Firmicutes, Bacteroidetes, Proteobacteria, Actinobacteria, and Verrucomicrobia, respectively, and after the intervention, they belonged to Firmicutes, Bacteroidetes, Actinobacteria, Proteobacteria, and Verrucomicrobia, respectively. It was observed that after the intervention, the relative abundances of Firmicutes (BT: 65.9%; PT: 69.2%) and Actinobacteria (BT: 1.8%; PT: 3.6%) increased, while the relative abundances of Bacteroidetes (BT: 28.0%; PT:23.8) and Proteobacteria (BT: 2.9%; PT: 1.8%) decreased. It was determined that the phylum Verrucomicribia (BT: 0.9%; PT: 0.7%) remained similar throughout the study period. No significant difference was observed at the phylum level after 8 weeks of intervention (*p* > .05).

**FIGURE 4:**
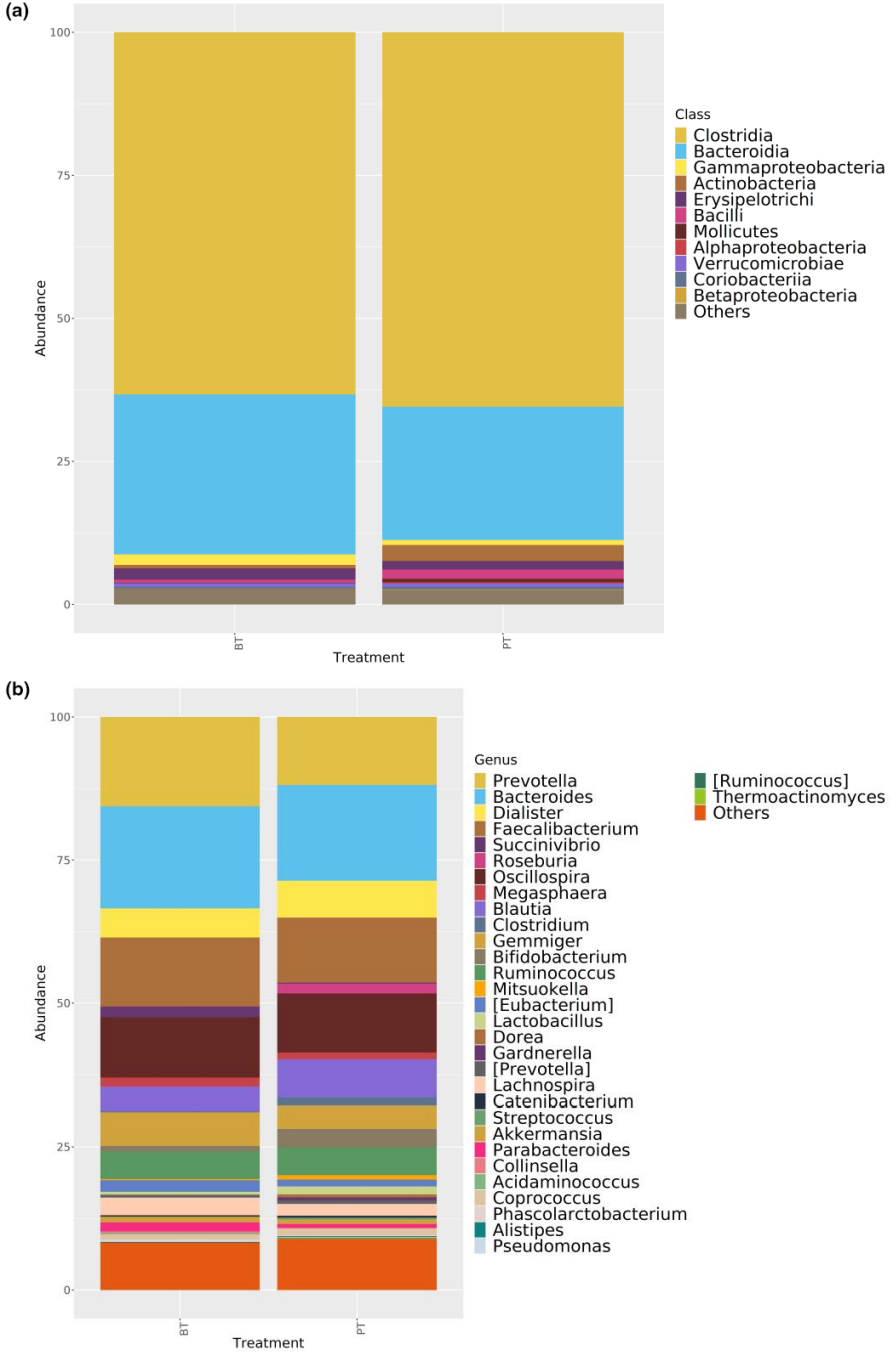
Intestinal microbiota composition at the class and genus levels. Bacterial community relative abundance analysis at the class (a) and genus (b) levels (relative abundance >1%; bacteria with relative abundance <1% were pooled in the “others” category and sorted by total concentration) (BT, before treatment; PT, post‐treatment).

Before and after the intervention, the class with the highest relative abundance, Clostridia (BT: 63.2%; PT: 65.8%), increased, and Bacteroidia (BT: 28.0%; PT: 23.6%), decreased. When other classes were examined, a decrease in the Gammaproteobacteria (BT: 2.3%; PT: 1.0%) class and an increase in the Actinobacteria (BT: 1.0%; PT: 2.8%) and Bacilli (BT: 0.8%; PT: 1.8%) classes were determined. The relative abundances of Erysipelotrichi (BT: 1.9%; PT:1.6%) and Verrucomicrobiae (BT:0.9%; PT:0.7%) remained similar. The change was found to be statistically significant for Bacilli from these classes (*p* < .05) (Figure 4a). The order of those with the highest relative abundance at the genus level is *Prevotella*, *Bacteroides*, *Faecalibacterium*, *Oscillospira*, *Gemmiger*, and *Dialister* before the intervention, and *Bacteroides*, *Prevotella*, *Faecalibacterium*, *Oscillospira*, *Blautia*, and *Dialister* after the intervention. At the genus level, the decrease in *Holdemania* and the increase in *Lactococcus* during the study period are statistically significant (*p* < .05) (Figure 4b).

**FIGURE 5:**
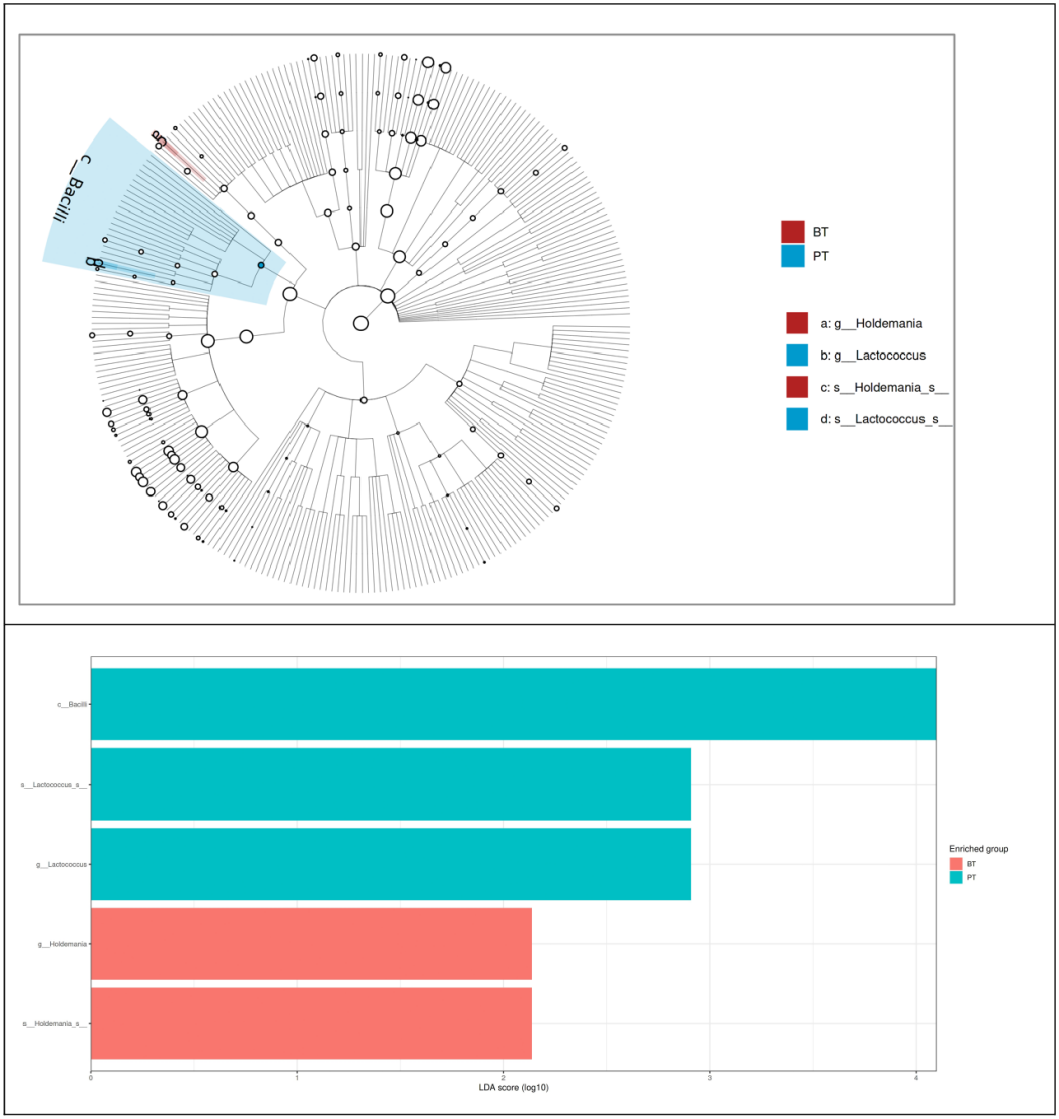
LEfSe analysis of participants before and after the intervention (BT, before treatment; PT, post‐treatment).

Figure 5 shows the results of the LEfSe analysis performed on the participants' fecal samples taken before and post‐treatment. After the treatment, the Bacilli class (*p* = .048) and its *Lactococcus* genus (*p* = .009) significantly increased; the *Holdemania* genus (*p* = .011) from the Erysipelotrichia class decreased (*p* < .05).

## 4 DISCUSSION

This study showed that kefir consumption provided some positive changes associated with the reduction of various symptoms in PCOS. In addition, this study is thought to be very important as it is the first study to evaluate the effects of kefir, which has been shown to have many health benefits in different disease groups, on PCOS.

Quality of life is significant for well‐being and health (Trent et al., 2002). PCOS has significant effects on the quality of life, and the deterioration in their quality of life seems remarkable compared to healthy women (Moreira et al., 2013). Relatedly, in a study conducted with 440 women with PCOS in Pakistan, SF‐12 was used to evaluate the quality of life of women. The majority of patients (85%) exhibited low quality‐of‐life scores, and depression was identified as the factor most contributing to low quality‐of‐life scores (Sidra et al., 2019). In this study, individuals' quality of life was evaluated with SF‐36. Accordingly, when the quality of life of PCOS was examined by subcategories, it was found that physical function and mental health scores showed a statistically significant improvement after the intervention (Table 1). This is a positive result; however, it is still thought that randomized controlled studies are needed to reveal this effect more clearly.

The health risks associated with PCOS extend beyond physical health (Alur‐Gupta et al., 2024). It shows the development of mental health problems with PCOS (Alur‐Gupta et al., 2024; Hu et al., 2024), and this situation can aggravate the pathological process of PCOS and make treatment difficult (Hu et al., 2024). A meta‐analysis on the subject emphasized the prevalence of anxiety and depression in PCOS, which suggests that there is a need to raise awareness about the psychosocial needs of women with PCOS (Yin et al., 2021). Fermented foods may be a treatment strategy for mental health problems such as depression or anxiety (Aslam et al., 2020). When some studies on physical function were examined, it was seen that physical function scores were higher in individuals who were more compliant with the energy‐restricted Mediterranean diet (Galilea‐Zabalza et al., 2018) or after essential amino acid supplementation (Rondanelli et al., 2011). A prominent component of the Mediterranean diet and a source of antioxidants, kefir also contains essential amino acids. Due to these properties, it may have increased physical function in this study.

Having antioxidant activity, kefir can be considered a potential candidate for beneficial natural antioxidant supplementation in humans (Liu et al., 2005; Vieira, de Sousa, et al., 2021; Vieira, Rosario, et al., 2021). During the microbial fermentation of kefir, several organic compounds, such as bacteriocins, bioactive peptides, antibiotics, exopolysaccharides, hydrogen peroxide, ethanol, carbon dioxide, and other vitamins (B1, B12), as well as amino acids and calcium peptides, are produced (Saleem et al., 2023). Thanks to these components, kefir is associated with several nutraceutical benefits such as antioxidative, antidiabetic, anticancer, antimicrobial, antihypercholesterolemic, and antihypertensive (Azizi et al., 2021). Kefir also exhibits anti‐inflammatory activity by inhibiting the activity of pro‐inflammatory cytokines like IL‐6, TNF‐α, and IL‐1β (Hamida et al., 2021). A recently published systematic review including 23 studies also showed that kefir consumption reduces inflammation through the switch between T helper (Th)1 and Th2 responses, decreasing the levels of pro‐inflammatory cytokines and increasing the levels of anti‐inflammatory cytokines (Albuquerque Pereira et al., 2024).

Gooruee et al. (2023) evaluated the effect of kefir intervention on inflammation in COVID‐19 patients using CRP and Erythrocyte Sedimentation Rate (ESR) markers. Consuming kefir twice a day (250 mL each time) for 2 weeks resulted in a significant decrease in ESR but did not significantly affect CRP (Gooruee et al., 2023). In a study examining TAS and TOS levels, 6 weeks of kefir consumption did not cause a significant change in the TOS levels of healthy men, but it significantly increased the TAS levels (Diken et al., 2022). Similar findings have been demonstrated in animal studies. Spontaneously hypertensive rats were divided into 3 groups to evaluate the effect of kefir group receiving 1 mL kefir/day (those with metabolic syndrome), positive control receiving 1 mL saline solution/day, and negative control receiving 1 mL saline solution/day (those without metabolic syndrome), and the rats were followed up with standard feed and water ad libitum for 10 weeks. A decrease in IL‐1β and an increase in IL‐10 were observed in the kefir group compared to the positive control. A decrease in oxidation products like hydroperoxides and MDA was also noted in the kefir group (Rosa et al., 2016). In hyperglycemic Wistar rats, plain kefir supplementation caused a statistically significant decrease in IL‐6 and IL‐1; a non‐significant decrease in TNF‐α; and an increase in IL‐10 (Hadisaputro et al., 2012). In another study in rats, lower IL‐6, TNF‐α, and IL‐1β expression and higher IL‐10 were observed in the group given kefir in addition to experimental periodontitis, compared to experimental periodontitis (Vieira, de Sousa, et al., 2021; Vieira, Rosario, et al., 2021). In the studies conducted on different groups of rats, TNF‐α levels were found to be lower in groups treated with kefir (Ekici et al., 2022; Sunita et al., 2023). In this study, while a statistically non‐significant decrease was observed in MDA, TOS, and TNF‐α (*p* > .05), the decrease in IL‐6 was found to be statistically significant (*p* < .05) (Table 2).

Some studies on the effect of kefir on the microbiota also seem promising. For example, it has been shown that kefir application can improve intestinal health in critically ill patients (Gupta et al., 2024). In a study conducted on mice, oral administration of kefir in alcoholic liver disease model mice, in addition to reducing inflammatory markers and increasing antioxidant levels, also regulated the gut microbiota composition (Cui et al., 2024). Similarly, a 4‐week kefir intervention was also associated with some positive changes in mice. Kefir increased catalase, superoxide dismutase (colon), and SCFAs in feces (butyrate) and the brain (butyrate and propionate). It has been shown that kefir contributes to the protection of intestinal and brain health by positively affecting the gut‐microbiota‐brain axis (Albuquerque Pereira, Morais de Ávila, et al., 2023). Similar results have also been supported by different studies (Albuquerque Pereira, de Ávila, et al., 2023; Ye et al., 2023).

In this study, participants' Bacilli class (*p* = .048) and its *Lactococcus* genus (*p* = .009) significantly increased after the intervention; the *Holdemania* genus from the Erysipelotrichia class (*p* = .011) decreased (*p* < .05) (Figure 5). Bacilli enter the gastrointestinal tract with probiotic preparations, water, and food (Ilinskaya et al., 2017). The ability to produce numerous antimicrobial compounds, secretory proteins, enzymes, carotenoids, and vitamins indicates the significance of Bacilli in the food chain (Elshaghabee et al., 2017). The genera *Bacillus* and *Lactobacillus*, two representatives of the Bacilli class, can considerably affect both the whole body and the intestinal microbiota because of the wide range of compounds they secrete despite their small proportions in the microbiome composition (Ilinskaya et al., 2017). Another genus of this class, *Lactococcus* species, is widely found in fish, other animals, and plants and is the normal biota of the gastrointestinal tract. They are accepted as beneficial and harmless because of their probiotic properties (Onyeaka & Nwabor, 2022). *Lactococcus*, together with genera like *Leuconostoc*, *Lactobacillus*, *Bifidobacterium*, and *Streptococcus*, constitute LAB and are found in fermented foods, especially fermented milk products (Linares et al., 2017). Using the *Lactobacillus plantarum* MA2 strain isolated from Tibetan kefir, Wang et al. (2009) added this strain to the high‐cholesterol diets of rats, and as a result, they revealed an increase in the fecal bifidobacteria and LAB population (Wang et al., 2009). It has been reported that PCOS was alleviated by regulating the intestinal microbiota as a result of the application of certain strains of Bifidobacterium and Lactobacillus to rats, in which a PCOS model was created with letrozole for 4 weeks (He et al., 2020).

Metabolites of lactic acid bacteria are significant for the intestinal microbiota (Śliżewska et al., 2020). These bacteria provide antihypertensive, anti‐inflammatory, antioxidative, immunomodulatory, antidiabetic, and anticholesterolemic effects on the consumer and microbiome modulation (Linares et al., 2017). In a study showing that the *Lactococcus* genus associated with these positive effects was reduced in PCOS patients, the abundance of the *Lactococcus* genus was reported to be significantly lower in non‐obese women with PCOS compared to healthy women (Zhou, Ni, Cheng, et al., 2020). Related to all this, kefir, along with the *Lactococcus* content, may increase the relative abundance of this genus in PCOS patients, thus creating many positive health effects, especially microbiota modulation. As in the literature (Kesmen & Kacmaz, 2011; Zanirati et al., 2015), the presence of *Lactococcus lactis* in the kefir used in this study may have caused the increase in the *Lactococcus* genus after kefir consumption.

*Holdemania*, on the other hand, is in the form of anaerobic, non‐spore‐forming rods and is a genus belonging to the Erysipelotrichaceae family and Erysipelotrichia class (Willems, 2015). However, there is limited information about the *Holdemania* genus in the literature, and it is stated that its health effects are not clear (Surono et al., 2020). The family of this genus, Erysipelotrichaceae, has been reported to be enriched in inflammation‐related diseases of the gastrointestinal tract (Kaakoush, 2015). Chen et al. (2012) showed that the abundance of the Erysipelotrichaceae family was increased in colorectal cancer patients compared to healthy controls (Chen et al., 2012). In addition, in mice that developed transmural inflammation similar to another disease, TNF‐induced Crohn's disease, the authors reported significant increases in Erysipelotrichaceae abundance (Schaubeck et al., 2016). In another study, the authors compared HIV‐uninfected controls and patients with chronic HIV infection receiving suppressive antiretroviral therapy. In conclusion, they observed that the relative abundance of Erysipelotrichi was positively correlated with TNF‐α levels (Dinh et al., 2015). Based on this, it can be said that the significant decrease in the *Holdemania* genus after the intervention may have created a positive microbiota composition change through inflammation; however, all genera belonging to a family or class cannot have similar characteristics. In fact, it should not be ignored that although many breeds are cited with negative or positive characteristics in the literature, there may also be studies showing the opposite.

## 5 CONCLUSIONS

As a result, to our knowledge, this was the first study exploring the effect of kefir on women with PCOS. This study indicated that consuming 250 mL of traditional kefir per day for 2 months could provide some positive changes in PCOS. Quality of life in the mental health and physical function categories significantly increased, and IL‐6 significantly decreased. Regarding microbiota composition, the relative abundance of the Bacilli class and its *Lactococcus* genus significantly increased, while the *Holdemania* genus decreased at the end of the study. The main treatment step for PCOS is lifestyle change, and based on these results, we think that kefir added to the diet may help reduce the risks of late‐term complications of PCOS by providing positive health outcomes. Further studies are needed to clarify the effectiveness of kefir on the microbiota and various health outcomes of women with PCOS, considering the limitations of this study.

### 5.1 Strengths and limitations

Information on the microbiota composition of the kefirs used in the studies is not presented for each study, so it is not possible to talk about standardization among all studies. Also, it is clear that the type and amount of microorganisms contained in kefir, the study population, and the study duration are factors that can directly affect the study results. The strengths of this study include the use of traditionally produced kefir and the knowledge of the diversity of microorganisms it contains. Therefore, these confounding factors should be considered when planning future studies and interpreting the study results.

## AUTHOR CONTRIBUTIONS

**Canan Yılmaz:** Methodology (equal); supervision (equal). **Merve Esra Çıtar Dazıroğlu:** Conceptualization (lead); data curation (lead); formal analysis (lead); methodology (lead); writing – original draft (lead). **Münire Funda Cevher Akdulum:** Methodology (equal); supervision (equal). **Ayşe Meltem Yalınay:** Methodology (equal); supervision (equal). **Nilüfer Acar Tek:** Conceptualization (equal); methodology (equal); supervision (lead); writing – review and editing (lead).

## FUNDING INFORMATION

This research was funded by the Gazi University Scientific Research Project TDK‐2022‐7864. The authors express thanks to Türkiye Bilimsel ve Teknolojik Araştırma Kurumu (TUBITAK) for providing open‐access publication fee.

## CONFLICT OF INTEREST STATEMENT

The authors declare no conflict of interest.

## Data Availability

The data that confirm the results of this research are available from the corresponding author upon reasonable request.
